# Tibetan Medical Formula Shi-Wei-Gan-Ning-Pill Protects Against Carbon Tetrachloride-Induced Liver Fibrosis – An NMR-Based Metabolic Profiling

**DOI:** 10.3389/fphar.2018.00965

**Published:** 2018-08-29

**Authors:** Xin Feng, Ming-Hui Li, Jing Xia, Da J. Deng Ba, Ling-Yu Ruan, Yue-Xiao Xing, Cheng Chen, Jun-Song Wang, Ge-Jia Zhong

**Affiliations:** ^1^Institute for Tibetan Medicine, China Tibetology Research Center, Beijing, China; ^2^Center for Molecular Metabolism, Nanjing University of Science and Technology, Nanjing, China; ^3^Division of TCM and Natural Products, Shanghai Institute for Food and Drug Control, Shanghai, China

**Keywords:** Tibetan medical formula, Shi-Wei-Gan-Ning-Pill, carbon tetrachloride, liver fibrosis, NMR, metabolic profiling

## Abstract

Liver fibrosis is a severe health problem, threatening the life quality and causing death, raising great concerns worldwide. Shi-Wei-Gan-Ning-Pill (SWGNP) is a traditional Tibetan recipe used to treat hepatic injuries; however, its hepatoprotective mechanism has not yet fully clarified. In this study, histological staining, biochemical assays, and elements determination were applied to evaluate the anti-fibrotic efficacy of SWGNP on a carbon tetrachloride (CCl_4_) induced hepato-fibrosis rat model. NMR-based metabolomics combined with orthogonal partial least squares-discriminant analysis (OPLS-DA), canonical regression analysis, and correlation networks analysis was used to characterize the potential biomarkers as well as metabolic pathways associated with the hepatoprotective activity of SWGNP. The results showed that SWGNP could significantly attenuate the pathological changes and decrease the levels of fibrosis markers (ColIV, HA, LN, and PCIII), and regulate the disordered elements distribution. Multivariate analysis and correlation network analysis revealed that SWGNP could protect rats against CCl_4_-induced liver fibrosis through anti-oxidation, repairing the impaired energy metabolisms and reversing the disturbed amino acids and nucleic acids metabolisms. In conclusion, this integrated metabolomics approach provided new insights into the mechanism of the hepatoprotective effect of SWGNP in liver fibrosis disease.

## Introduction

Hepatic fibrosis, characterized by excessive accumulation of extracellular matrix (ECM; Kaffe et al., 2017), is a serious disease caused by acute or chronic liver injuries such as viral infection, poison agents invasion, excessive drinking, cholestasis, etc. (Bangen et al., 2017). With the advancement of fibrosis, formation of fibrous septa and distortion of liver architecture appear, which will impair normal liver functions and eventually shift to an end stage of cirrhosis and/or hepatocarcinoma (Pellicoro et al., 2014). Growing evidences suggest that progressive liver fibrosis is a multifactor disease (stress molecules, metabolic toxins, cytokines, growth factors, etc.) *via* multiple mechanisms and pathways. Due to the complexity of this disease, liver fibrosis still remains an unconquered disease and a challenging task, especially for western medicine, which embraces one drug/target for one disease.

Differing from western medicine both in philosophy and methodology, the traditional Tibetan medicine has its own advantages in the treatment of liver disease, which prefer combination therapies that comprise more than one active ingredients (Qiu, 2007). Characterized by holistic theory, multi-link and component therapeutics, Tibetan medicine can hit multiple targets simultaneously and exert synergistic therapeutic efficacies, which offers bright prospects and superior advantages in the treatment of liver injury. Shi-Wei-Gan-Ning-Pill (SWGNP), an empirical Tibetan formula, was developed by a great master of Tibetan medicine called Cuo-Ru-Ci-Lang (1928–2004) based on the substantial clinical practice and experience. SWGNP has been used to treat various liver injuries including fatty liver, viral hepatitis, hepatic fibrosis, hepatocirrhosis, and hepatocarcinoma with evident efficacy in clinic, which has a regulating function on “ci ba jiu xie” in Tibetan medicine theory. However, the underlying mechanisms of SWGNP in its treatment of liver fibrosis have still not been fully evaluated as yet.

Metabolomics represents a holistic and systemic perspective of endogenous metabolites profiling in organisms, which inspect the changes of small-molecule (molecular weight less than 1000) metabolites that varying in response to the pathophysiology of the body, mutations in genes and enzymes, and internal and external environment status (Samuelsson et al., 2006; Baker, 2011). Metabolomics is highly sensitive in detecting drug effects, because the perturbations of metabolic profiling usually happened much earlier than the histopathological alterations, which makes it especially appropriate for the assessment of the comprehensive effects of herbal medicines. Nuclear magnetic resonance (NMR) technique has become a useful analytical platform in metabolomics research for its high throughput, non-bias, simple sample preparation, non-destructive nature, and rich in structural information.

In this study, the anti-fibrotic efficacies of SWGNP on a CCl_4_-induced hepatic fibrosis rats model and potential mechanisms were investigated using metabolomics combined with histopathological inspection, biochemical assays and elements. SWGNP could effectively relieve hepatic pathology of fibrosis rats and reverse CCl_4_ induced severe metabolic disorders.

## Materials and Methods

### Materials

The SWGNP formulae consist of 12 Tibetan medicines, including seven botanical medicines such as *Carthamus tinctorius, Crocus sativus, Herpetospermum caudigerum, Meconopsis integrifolia, Dracocephalum tanguticum, Saxifraga stolonifera, Corydalis impatiens*, and five mineral medicines such as Bear Gallbladder, Calculus Bovis, Brag-zhun, Calciosinti, and Turquoisis. The medicines of the formulae SWGNP were purchased from Jiangsu Medical Company (Nanjing, China) and authenticated by a pharmacist. The commercial kits used for biochemical assays were obtained from Nanjing Jiancheng Bioengineering Institute (Nanjing, China). Other chemical regents were purchased from Sigma (St. Louis, MO, United States).

First, all the botanical medicines except *C. sativus* were extracted with 95% ethanol (1:8, 1:8, and 1:5, w/v) under reflux for three times (3, 3, and 1 h). The decoction was concentrated to extractum using a rotary evaporator, and then stored at 4°. Second, the five mineral medicines as well as *C. sativus* were ground to powder. Third, blended the extractum with the powder, suspended the mixture with 0.3% carboxymethyl cellulose sodium solution before intragastric administration. HPLC-Q-TOF-MS method was used to identify the chemical constituents of SWGNP extracts (**Supplementary Table S1**).

### Animal Experiments

Protocols for animal studies were approved by the Animal Policy and Welfare Committee of South-central University for Nationalities. Healthy male Wistar rats in a SPF grade, weighing about 200–240 g at six to eight weeks of ages, were obtained from Liaoning Changsheng Biotechnology Co., Ltd., license NO: SCXK(Liao) 2015-0001. Animals were housed in individual ventilated cages under controlled conditions (22°C, relative humidity 45–70%, 12/12 h light/dark cycle), with free access to regular chow food and water, license NO: SYXK(E) 2016-0089. The animals were acclimatized to the laboratory for seven days before experiment.

Rats were divided into five groups of 15 animals each: normal control, model, and three doses of SWGNP groups: low (63 mg/kg, SWGNP-L), medium (126 mg/kg, SWGNP-M), and high (252 mg/kg, SWGNP-H). The daily dose of SWGNP for patients was 20.96 mg/kg in clinic. Hence, the dose ranges in our study were therapeutically relevant. From week 1 to week 3, rats in the normal control group were intraperitoneally injected with olive oil once every 3 days. Rats in other groups were peritoneally injected with CCl_4_ blended with olive oil (1:1, v/v) at the dose of 3 mL/kg once every 3 days, lasting for 7 weeks throughout the experiment to establish the liver fibrosis model. From week 4 to week 7, rats in the SWGNP treatment groups were orally administrated with different doses of SWGNP with the volume of 10 mL/kg/day (suspended in 0.3% CMC-Na). Rats in the control and model groups were received only the vehicle from week 4 to week 7 with the volume of 10 mL/kg/day (0.3% CMC-Na, p.o.). All animals were weighed once a week.

At the end of week 7, rats were fasted for 12 h after the last time of administration, weighted, and anesthetized with pentobarbital. Blood samples were collected from the abdominal aorta and serum were obtained after centrifugation at 3000 rpm, 4°C for 10 min, liver tissues were harvested and stored at -80°C, and livers indexes (liver index = weight of tissues/body weight × 100%) were calculated.

### Hematoxylin-Eosin and Masson Staining

Liver tissues were randomly taken from the right, median, and left lobes, washed with cold phosphate buffer (pH 7.4), and then fixed in 10% neutral-buffered formalin solution for 24 h. They were dehydrated in graded ethylic alcohol and embedded in paraffin, which were then sliced into 5 μm thin-sections along the largest truncation surface (*N* = 4). The slices were stained with hematoxylin-eosin (HE) and Masson trichrome for histological examination under light microscopy.

The semi-quantitative fibrosis staging scores were assessed according to previous report: 0: normal; 1: slight, fibrosis located in the center of the hepatic lobule; 2: moderate, fibrous space formation, but the hepatic lobule structure reserved; 3: severe, fibrous space enlarged, and hepatic lobular structure distorted; 4: early or certain cirrhosis, pseudo-lobule formation.

### Biochemical Assays

Liver function markers such as glutamic-pyruvic transaminase (GPT), glutamic-oxalacetic transaminase (GOT), albumin (ALB), and total bilirubin (TBil), oxidative stress parameters such as superoxide dismutase (SOD), glutathione peroxidase (GPX), catalase (CAT), and alonyldialdehyde (MDA), and hepatic fibrosis markers such as hyaluronidase (HA), laminin (LN), type III procollagen (PCIII), and type IV collagen (ColIV) were measured using the commercially available kits (Nanjing Jiancheng Biotech Inc., Nanjing, China) according to the manufacturer’s instructions.

### Inductively Coupled Plasma Mass Spectrometer

Iron concentrations of calcium (Ca), magnesium (Mg), aluminum (Al), zinc (Zn), iron (Fe), copper (Cu), arsenic (As), phosphor (P), and potassium (K) in liver tissues and serums were determined with an Agilent 7500ce inductively coupled plasma mass spectrometer (ICP-MS, Agilent 7500ce, Agilent Technologies, Santa Clara, CA, United States) by Institute for Nutritional Sciences, Shanghai Institute of Life Sciences, Chinese Academy of Sciences (Shanghai, China).

### Sample Preparation and ^1^H NMR Analysis

One aliquot of liver tissues was weighed, homogenized with 50% acetonitrile (1:5, w/v) bathing in ice. After vortex and centrifugation (12,000 rpm, 4°C for 10 min), the supernatants were carefully collected and transferred into fresh tubes, frozen and lyophilized to dryness. The dried samples were stored at -80°C prior to analysis. Frozen serum samples were thawed at room temperature and 500 μL of each was added with 1 mL methanol to precipitate proteins. The mixtures were vortexed at -20°C for 20 min, then centrifuged to obtain supernatants at 13,400 rpm for 30 min. The supernatants were decanted into fresh vials and lyophilized, stored at -80°C prior to analysis. For NMR tests, samples were dissolved in 550 μL 99.8% D_2_O phosphate buffer (0.2 M, pH = 7.0) containing 0.05% (w/v) sodium 3-(trimethylsilyl) propionate-2, 2, 3, 3-d4 (TSP). After vortexed for 15 s and centrifuged at 12,000 rpm, 4°C for 10 minutes, the supernatants were transferred to a clean NMR tubes (5 mm) for analysis.

^1^H NMR spectra were recorded on a 500 MHz NMR spectrometer (Bruker AVANCE III) at 298 K. D_2_O was used for field frequency locking with TSP for chemical shift reference (^1^H, δ 0.00). A transverse relaxation-edited Carr-Purcell-Meiboom-Gill sequence [90(τ-180-τ) n-acquisition] with a total spin-echo delay (2 nτ) of 40 ms was employed. The spectra were recorded with 128 scans into 64 K data points over a spectral range from -5 to 15 ppm. The spectra were Fourier transformed by multiplication of the FIDs with an exponential weighting function corresponding to a line-broadening of 0.5 Hz.

### Multivariate Analysis

After phase and baseline correction and zero point alignment using the Topspin software (Topspin 3.5, Bruker), the NMR spectra were converted to text files using MestReNova software (version 11, Mestrelab Research SL), and then imported into R software for data processing. After removal of the residual water signals (4.54–5.18 ppm), spectra were binned using an adaptive, intelligent algorithm (De Meyer et al., 2008). Data were probabilistic quotient normalized (Dieterle et al., 2006) and Pareto scaled before multivariate analysis.

The supervised orthogonal partial least squares-discriminant analysis (OPLS-DA) was performed using home made R code to maximize covariance between the metabolome data and the predictive classifications. The clusters between groups were displayed in the scores plots. Loadings plot was used to seek for differential metabolites between classes. The differential metabolites were color coded according to the absolute values of correlation coefficients from blue (low coefficients) to red (high coefficients). The OPLS-DA model was validated by a repeated twofold cross validation (2-CV) method and a permutation test. The total explained variations of the model were evaluated by R^2^Y, and the model predict ability was evaluated by Q^2^Y.

Metabolites were identified by searching the public metabolome databases such as Madison-Qingdao Metabolomics Consortium Database (Cui et al., 2008) and Human Metabolome Database (Wishart et al., 2012). Some auxiliary methods such as the statistical total correlation spectroscopy techniques (Cloarec et al., 2005) and the commercial available software Chenomx NMR suite (Chenomx Inc., Edmonton, Canada) were also used for metabolites identification.

### Univariate Analysis

According to the conformity to normal distribution of the variables, parametric student’s *t*-test or non-parametric rank test was applied to examine the significance of the metabolites changes between groups. The fold change values of metabolites and the associated *P*-values corrected by Benjamini–Hochberg adjusted method (Hochberg and Benjamini, 1990; Benjamini and Hochberg, 1995) were calculated and visualized as colored tables.

### Canonical Regression Analysis

To excavate the correlations between metabolites, elements and biochemical parameters, the data (**Tables 1, 2**) were subjected to canonical regression analysis with metabolite concentrations as X variables and other parameters as Y variables.

**Table 1:** Potential marker metabolites in rat liver extracts identified by 1H-NMR and their 530 variations among groups and the associated *p*-values.

Compounds	Model/control		Low/model		Medium/model		High/model	
	log_2_(FC)^*a*^	*p*^*b*^	log_2_(FC)	*p*	log_2_(FC)	*p*	log_2_(FC)	*p*
Hypoxanthine	–1.11	^∗∗∗^	0.06		–0.32		–0.43	^∗∗^
Inosine	–1.06	^∗∗∗^	0.7	^∗∗^	–0.12		1.25	^∗∗∗^
Xanthine	–1.04	^∗∗∗^	0.06		–0.32		–0.43	^∗∗^
Fumarate	–0.96	^∗∗∗^	0.11		0.38		0.44	^∗∗^
Glucose	–0.77	^∗∗∗^	0.07		0.01		–0.17	
Niacinamide	–0.68	^∗∗^	–0.12		–0.04		–0.07	
NADPH	–0.57	^∗∗^	0.26	^∗^	–0.01		0	
3-Hydroxybutyrate	–0.57	^∗∗^	0.14		–0.18		0.01	
Tyrosine	–0.43	^∗∗^	–0.07		–0.07		–0.76	^∗∗∗^
Histidine	–0.37		–0.14		0.19		0.04	
NAD^+^	–0.28		0.39		–0.03		0.28	
Isoleucine	–0.23		0.06		–0.17		–0.47	^∗∗∗^
Lysine	–0.21	^∗∗^	–0.22		–0.15		–0.45	^∗∗∗^
Leucine	–0.19		–0.06		–0.18		–0.67	^∗∗∗^
Creatine	–0.17		–0.12		–0.09		–0.04	
Choline	–0.14		0.2		–0.12		–0.22	
Acetate	–0.1		0.12		–0.07		0.18	^∗^
Valine	–0.09		–0.03		–0.18		–0.69	^∗∗∗^
Phenylalanine	0.05		0.04		–0.03		–0.81	^∗∗∗^
Betaine	0.11		–0.07		0.3		0.08	
5,6-Dihydrouracil	0.15		0.18		0		0.06	
Glycine	0.27		–0.18		–0.3		–0.4	^∗∗^
Glutamate	0.29		–0.15		–0.09		–0.17	
Uridine	0.29	^∗^	0.26		0.04		0.42	^∗^
Uracil	0.33	^∗^	–0.14		0.29		–0.25	^∗^
Aspartate	0.33		–0.29		–0.17		–0.27	^∗^
Ethanol	0.39		–0.94	^∗∗∗^	–0.56	^∗^	–1.22	^∗∗∗^
Glutamine	0.43	^∗∗^	0.15		0.06		0.37	^∗∗∗^
Alanine	0.49	^∗∗∗^	–0.09		–0.12		–0.3	^∗^
Dimethylamine	0.52	^∗∗^	–0.22		–0.17		–0.15	
UDP-galactose	0.53	^∗∗^	0.41		0.15		0.67	^∗∗∗^
Glutathione	0.54	^∗^	0.03		–0.06		0.32	^∗∗^
Succinate	0.57	^∗∗^	–0.14		–0.3		–0.2	
*sn*-Glycero-3-phosphocholine	0.59	^∗∗∗^	0.1		0.16		0.2	^∗∗^
Sarcosine	0.63	^∗^	0.18		–0.04		0.06	
Phosphocholine	0.63	^∗∗^	–0.07		0.06		0.19	^∗^
Phosphoethanolamine	1.13	^∗∗∗^	0.04		0.87		0.74	
UDP-glucose	1.21	^∗∗^	0.43		0.08		0.97	^∗∗∗^
Lactate	1.23	^∗∗∗^	–0.2		0		–0.12	

*^*a*^FC: color-coded according to the fold-change value; red represents increased and blue represents decreased concentrations of metabolites.*

*^*b*^*P*-values corrected by Benjamini–Hochberg methods were calculated based on a parametric Student’s t test or a non-parametric Mann–Whitney test (dependent on the conformity to normal distribution). ^∗^*P* < 0.05, ^∗∗^*P* < 0.01, ^∗∗∗^*P* < 0.001.*

**Table 2:** Potential marker metabolites in rat serum identified by ^1^H-NMR and their variations among groups and the associated *p* values.

Compounds	Model/control		Low/model		Medium/model		High/model	
	log_2_(FC)^*a*^	*p*^*b*^	log_2_(FC)	*p*	log_2_(FC)	*p*	log_2_(FC)	*p*
Succinate	–0.74		–0.23		–0.16		–0.04	
GABA	–0.57		–0.08		–0.05		0.08	
3-Hydroxybutyrate	–0.46		–0.04		0.02		0.2	
Glucose	–0.45	^∗∗^	0.05		0.12		0	
Acetoacetate	–0.32		–0.4		–0.28		–0.77	
Tyrosine	–0.27		0.12		0.03		0.26	^∗∗^
Tryptophan	–0.19		0.1		–0.1		0.27	
3-Hydroxyisobutyrate	–0.17		–0.14		0.21		0.1	
Uridine	–0.03		–0.14		–0.01		0.05	
Formate	–0.02		–0.25		–0.27		0.04	
Inosine	–0.01		0.03		–0.06		0.1	
Creatine	0.00		–0.22		–0.23		–0.04	^∗∗^
Methanol	0.02		0.12		0.04		–1.12	
Phenylalanine	0.04		0.06		–0.09		0.18	
Lysine	0.06		–0.02		–0.19		0.13	
Pyruvate	0.08		0.49		–0.25		0.33	
Glutamine	0.18	^∗^	–0.08		–0.19		–0.03	
Acetate	0.27		–0.15		–0.28		0.13	
Citrate	0.31		0.02		–0.13		0.15	
Isoleucine	0.32	^∗^	–0.08		–0.24		0.11	
Valine	0.41	^∗∗^	–0.14		–0.27		0.1	
Ethanol	0.47		0.02		–0.13		0.15	
Lactate	0.53	^∗^	–0.08		–0.01		0.32	
Alanine	0.65	^∗^	0.04		–0.09		0.22	
Leucine	0.74	^∗∗^	–0.13		–0.23		0.08	
Glycine	0.82	^∗∗^	–0.06		–0.07		0.07	
Histamine	0.95		0.06		–0.07		0.13	
Acetone	1.01	^∗∗^	–0.42		–0.51		–0.32	

*^*a*^FC: color-coded according to the fold-change value; red represents increased and blue represents decreased concentrations of metabolites.*

*^*b*^*P*-values corrected by Benjamini–Hochberg methods were calculated based on a parametric Student’s *t*-test or a non-parametric Mann–Whitney test (dependent on the conformity to normal distribution). *^∗^*P < 0.05, ^∗∗^*P* < 0.01.*

### Correlation Network Analysis

Correlation-based networks of metabolome data were constructed (Langfelder and Horvath, 2008) according to the Pearson correlation coefficients of metabolites from all samples. Correlations of metabolites above a threshold (0.6) were connected each other by solid lines, color coded according to the absolute correlation coefficients (reddish and bluish color represented positive and negative correlations, respectively), with line width scaled according to the absolute values of correlation coefficients. The Pearson correlation networks were mapped onto the KEGG biochemical reaction networks (metabolites connected by gray lines) in order to attain a strong support for the metabolites Pearson correlation networks (Li et al., 2017).

## Results

### SWGNP Could Ameliorate Liver Histopathological Injuries of Fibrosis Rats

**FIGURE 1:**
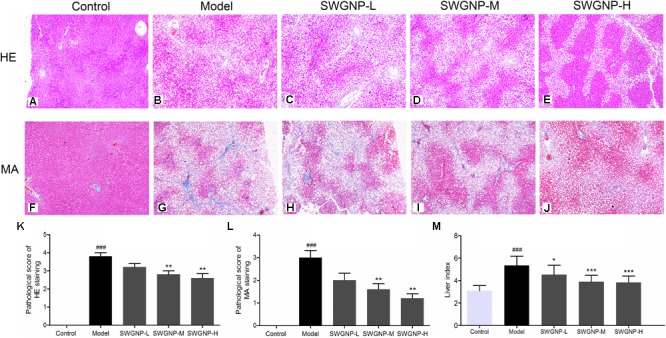
HE staining **(A–E)**, Masson staining **(F–J)**, and semi-quantitative analysis **(K–M)** of liver tissues. With HE staining (200x), the nuclear was shown blue and cytoplasm was red. With Masson staining (200x), the collagen fiber was shown blue and hepatic cells were red. Liver of control rats exhibiting no obvious pathological changes. Livers of CCl_4_ dosed rats showing severe fat vacuoles, significant hepatic cell necrosis, and excessive collagen deposition. SWGNP could decrease the pathological scores and liver indexes in a dose dependent manner. Livers after low dose of SWGNP administration showing severe fat vacuoles and collagen deposition. Livers after medium dose of SWGNP administration showing slight hepatic cell necrosis and less collagen deposition and fat vacuoles. Livers after high dose of SWGNP administration displaying with no obvious collagen deposition without any sign of cell degeneration or necrosis. Values were expressed as mean ± SD (*N* = 6). ###*p* < 0.001 vs. control rats; ^∗^*p* < 0.05, ^∗∗^*p* < 0.01, and ^∗∗∗^*p* < 0.001 vs. model rats.

Under the action of cytochrome P450 (CYP2E1), CCl_4_ was metabolized to highly reactive free radicals such as trichloromethyl and trichloromethyl peroxy (metabolism activation), which can lead to cell and mitochondrial membrane lipid peroxidation (Kaplowitz, 2000). Free radicals can also covalently binds to macromolecules, which caused severe hepatocyte injury and necrosis, and deposition of a large amount of collagen in the liver, leading to liver fibrosis. The HE (**Figure 1A**) and Masson (**Figure 1F**) stained rat livers in the control group showed normal lobular architecture with central veins and radiating hepatic cords. Intraperitoneal injection of CCl_4_ caused severe hepatic pathological damages such as the appearance of massive fat vacuoles, inflammation and significant hepatic cell necrosis (**Figure 1B**). The semi-quantitative hepatic HE staging score were 3.80 ± 0.44, 3.2 ± 0.45, 2.80 ± 0.45, and 2.60 ± 0.55 for model group and the low, medium, and high dose of SWGNP treatment group, respectively (**Figure 1K**). Masson staining for fibrous tissues confirmed the presence of fibrous septa and excessive collagen deposition in livers after chronic CCl_4_ intoxication (**Figure 1G**). The semi-quantitative hepatic fibrosis staging score was greatly increased to 3.00 ± 0.71 in the model group (*P* < 0.01 vs. control group). In contrast, livers from different doses of SWGNP-treated rats appeared with ameliorative architecture, less hepatic cells necrosis, less collagen deposition, with only a slight increase of reticular fibers **(Figures 1C–E,H–J)** and a significant dose-dependent decrease of the staging score of 2.00 ± 0.71,1.6 ± 0.55, and 1.2 ± 0.45 for low, medium, and high dose of SWGNP treatment (**Figure 1L**). CCl_4_ also induced significant increase of liver indexes, which could be decreased by SWGNP in a dose-dependent manner (**Figure 1M**). These findings suggest that liver fibrosis can be greatly ameliorated after SWGNP treatment.

### SWGNP Could Decrease the Liver Fibrosis Markers and Increase Antioxidant Activity

**FIGURE 2:**
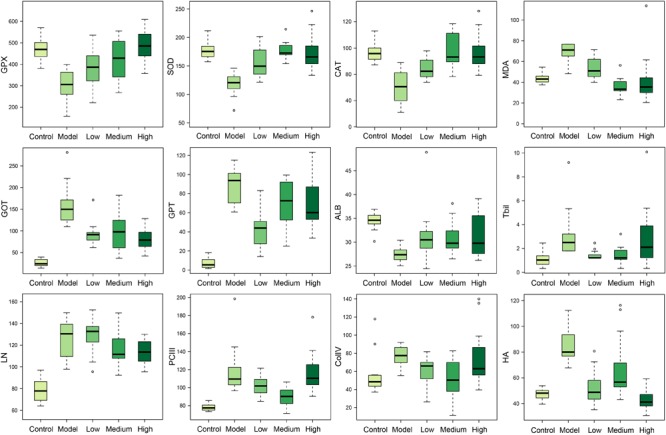
Boxplots of serum markers of oxidation stress (GPX, SOD, CAT, and MDA), liver function (GOT, GPT, ALB, and TBil), and hepatic fibrosis (LN, PCIII, ColIV, and HA) for rats form control, model, and low, medium, and high dose of SWGNP treated groups. The boxes cover 25% quartile and 75% quartile of the data. The line in the box represents the median value. The extended whiskers show the extent of the rest of the data, and outliers are shown as open circle.

The liver fibrosis markers such as ColIV, HA, LN, and PCIII were significantly increased in rats of the model group (**Figure 2**), demonstrating the formation of liver fibrosis. Located mainly in the liver, the two transaminases (GPT and GOT) could be released into the blood in case of liver injury. The increased level of TBil was an indicator of liver injury or biliary abnormality. ALB and ceruloplasmin are synthesized by liver; liver damage can lead to decreased levels of ALB and ceruloplasmin. In the model group, important antioxidant enzymes GPX, CAT, and SOD were significantly decreased and MDA, a product of lipid peroxidation, was markedly increased (**Figure 2**). The results reflected that CCl_4_ injection induced free radical damage and redox imbalance, which could be partially or fully restored to their normal status by SWGNP treatment.

### SWGNP Could Regulate the Elements Concentrations

**FIGURE 3:**
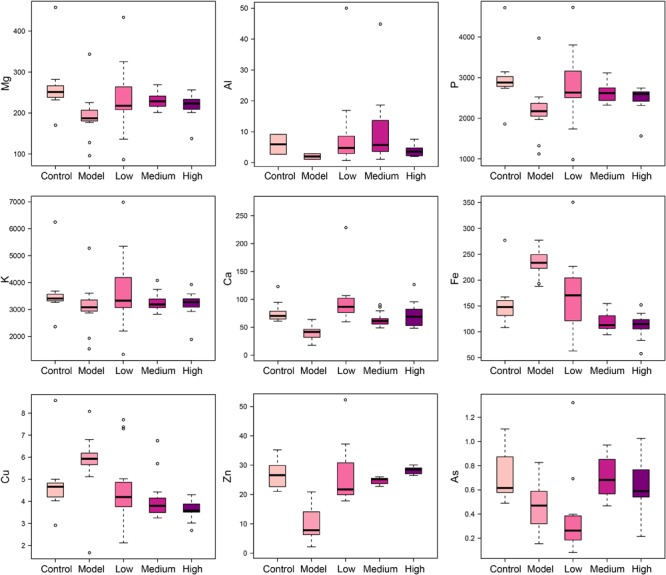
Boxplots of elements levels in liver tissues of rats form control, model, and low, medium, and high dose of SWGNP treated groups. The boxes cover 25% quartile and 75% quartile of the data. The line in the box represents the median value. The extended whiskers show the extent of the rest of the data, and outliers are shown as open circle.

**FIGURE 4:**
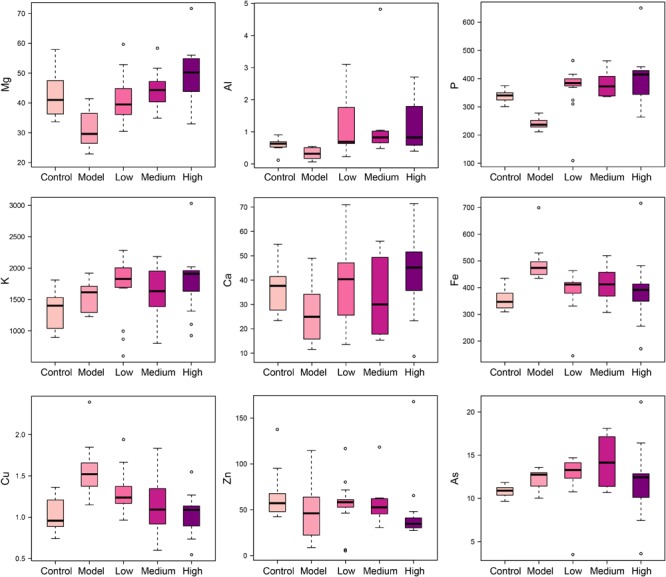
Boxplots of elements levels in serums of rats form control, model, and low, medium, and high dose of SWGNP treated groups. The boxes cover 25% quartile and 75% quartile of the data. The line in the box represents the median value. The extended whiskers show the extent of the rest of the data, and outliers are shown as open circle.

Elements are co-factors of many biological enzymes, and thus inextricably involved with various biochemical reactions in organisms. In liver tissues of model group, Mg, Ca, P, and Zn were significantly decreased, and Cu and Fe were significantly increased in livers and serum of fibrosis rats (**Figures 3, 4**). It has been demonstrated that Zn could prevent hepatic fibrosis by inhibiting lysyl oxidase which is involved in the intermolecular cross-linkage of collagen. In addition, Cu could promote hepatic fibrosis by acting as a cofactor of lysyl oxidase (Boyett and Sullivan, 1970). It has been reported that the copper content was increased in liver cirrhosis and liver carcinoma patients. Excess Fe ions are released into cytoplasm as free ions, which could promote oxidative stress and induce hepatocellular necrosis and collagen production ultimately. After SWGNP administration, the levels of these elements were significantly reversed, suggesting that SWGNP could prevent liver fibrosis through regulating the disturbed levels of elements. The possible role of SWGNP supplementation in regulating the elements concentrations may include reducing oxidative stress and increasing anti-oxidase activity, thus restored the function of hepatocytes, bringing the altered elements metabolisms back to normal.

### SWGNP Could Reverse the Disturbed Metabolisms Toward the Normal Status

**FIGURE 5:**
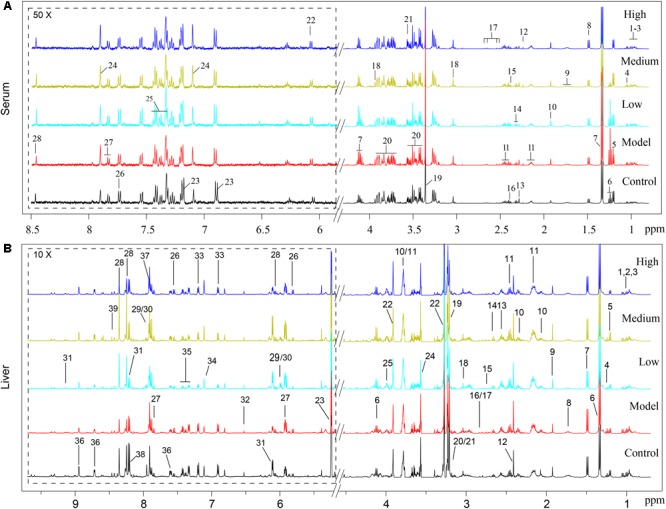
**(A)** Typical 500 MHz^1^H NMR spectra of the serums from rats of control, model, and low, medium, and high dose of SWGNP treated groups: 1. isoleucine, 2. leucine, 3. valine, 4. 3-hydroxyisobutyrate, 5. 3-hydroxybutyrate, 6. ethanol, 7. lactate, 8. alanine, 9. lycine, 10. acetate, 11. glutamine, 12. acetone, 13. acetoacetate, 14. GABA, 15. pyruvate, 16. succinate, 17. citrate, 18. creatine, 19. methanol, 20. glucose, 21. glycine, 22. inosine, 23. tyrosine, 24. histamine, 25. phenylalanine, 26. tryptophan, 27. uridine, and 28. formate. **(B)** Typical 500 MHz ^1^H NMR spectra of the liver extracts from control, model, and low, medium, and high dose of SWGNP-treated rats.1. Isoleucine, 2. leucine, 3. valine, 4. ethanol, 5. 3-hydroxybutyrate, 6. lactate, 7. alanine, 8. lysine, 9. acetate, 10. glutamate, 11. glutamine, 12. succinate, 13. glutathione, 14. 5,6-dihydrouracil, 15. aspartate, 16. dimethylamine, 17. sarcosine, 18. creatine, 19. choline, 20. phosphocholine, 21. *sn*-glycero-3-phosphocholine, 22. betaine, 23. glucose, 24. glycine, 25. phosphoethanolamine, 26. uracil, 27. uridine, 28. inosine, 29. UDP-glucose, 30. UDP-galactose, 31. NAD^+^, 32. fumarate, 33. tyrosine, 34. histidine, 35. phenylalanine, 36. niacinamide, 37. xanthine, 38. hypoxanthine, and 39. NADPH.

**FIGURE 6:**
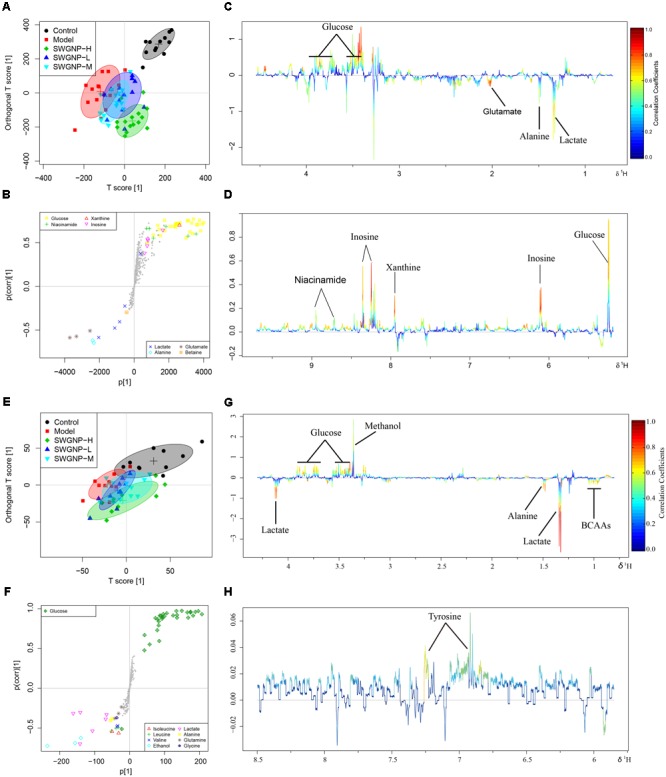
Multivariate analysis of ^1^H NMR data of liver **(A–D)** and serum **(E–H)** extracts for control, model, and low, medium, and high dose of SWGNP treated rats. Score plots **(A,E)** and corresponding S-/loadings plot **(B–D,F–H)** for OPLS-DA, color-coded with the absolute value of correlation coefficients.

Typical ^1^H NMR spectra of liver extracts and serums are shown in **Figure 5**, and the detailed metabolite assignments are listed in **Tables 1, 2**. Supervised OPLS-DA was performed on the binned ^1^H NMR metabolomics data to acquire an overview of variations among groups. In the scores plots for the liver, the CCl_4_ dosed groups were well separated from the control group (**Figure 6A**). Similar patterns were found in serum scores plots (**Figure 6E**). The overlapping between the low and medium dose SWGNP treatment group and the model group and, by instead, the furthest distance between high-dose SWGNP treatment group and model group, revealing a dose-dependent manner of SWGNP induced liver protection. In livers of the model group, levels of xanthine, inosine, hypoxanthine, glucose, fumarate, 3-hydroxybutyrate, NADPH, nicotinamide, tyrosine, and lysine were significantly decreased, and those of uridine, alanine, aspartate, glutamine, lactate, UDP-galactose, glutathione, succinate, *sn*-glycero-3-phosphocholine, UDP-glucose, dimethylamine, phosphoethanolamine, and phosphocholine, were significantly elevated (**Figures 6B–D**). The significantly decreased compounds in serum of model group were glucose, and the significantly elevated metabolites in liver of the model group were glutamine, isoleucine, leucine, valine, lactate, alanine, and glycine (**Figures 6F–H**). After SWGNP treatment, the above disturbed metabolites could be partially reversed.

### The Metabolites Changes Have Close Associations With Levels of Elements and Blood Parameters

**FIGURE 7:**
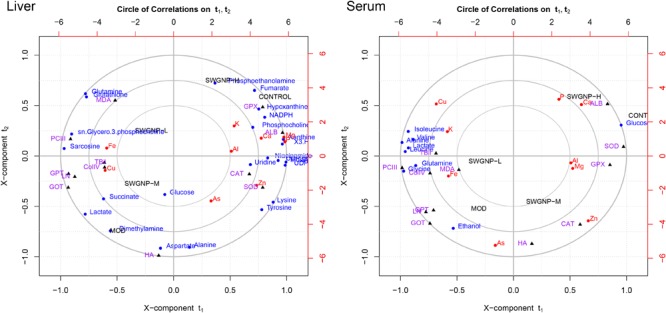
Relationships between metabolites, elements, and biochemical parameters as revealed by canonical regression analysis using metabolites concentrations as *x* variables and biochemical parameters and elements contents as *y* variables, with three circles from inner most to outer most denoting radii of 0.50, 0.75, and 1.00, respectively.

In order to seek the correlations between the development of hepatic fibrosis and the alterations of metabolome, minerals, and blood parameters, the canonical regression analysis was performed with metabolite concentrations as X variables and the biochemical parameters and elements as Y variables. In the biplot, the control and model groups were separated far away along X-component t1, with the SWGNP treated groups in the between (**Figure 7**).

Some interesting correlations between these data were found. Energy substances such as glucose and lactate were negatively correlated with liver function (GOT and GPT) and positively with fibrosis markers (ColIV, LN, PCIII, and HA), indicating that energy metabolism was shifted from aerobic into anaerobic respiration in liver fibrosis rats. The membrane-related compounds, *sn*-glycero-3-phosphocholine, and the antioxidant glutathione were positively correlated with the liver function and fibrosis markers, proved that oxidative stress occurred in liver fibrosis rats.

Albumin and Ca were significantly correlated with each other in the correlation circle plot. The results of biochemical and elemental assays showed that significantly decreased levels of ALB and Ca were observed. The marked decrease of serum ALB levels may be partly responsible for the reduced serum calcium levels because about 47% of serum calcium is bound to proteins, especially ALB (George, 2006).

Lipids related metabolites such as phosphoethanolamine and phosphocholine were significantly correlated with Zn in the correlation circle plot. The blood zinc concentration in the fibrosis rats is obviously reduced. Zinc is involved in stabilization of the cell membrane and prevention of oxidative destruction caused by free radicals. Zn deficiency is usually associated with reduced free radicals clearance capacity and increased lipid oxidation, leading to decrease of collagenase activity (Giménez et al., 1994). The positive correlation between Zn and phosphoethanolamine and phosphocholine manifested that they were associated with liver dysfunction.

Cu and Fe were significantly correlated with MDA, GOT, GPT, PCIII, ColIV, and LN in the correlation circle plot. Levels of Cu and Fe were significantly increased in fibrosis rats. The increased levels of Cu and Fe could promote the synthesis of collagen because Fe is needed for the hydroxylation of procollagen α-peptide chain, and Cu is the auxiliary factor of lysyl-oxidase which is the key enzyme for bridging collagen molecules to form collagen fibers. The accumulation of copper in liver and serum may be attributed to the destruction of the bile duct and the hepatocytes, leading to obstructed copper excretion. The increased Fe in fibrosis rats may be attributed to portal hypertension, iron malabsorption caused by intestinal congestion and loss of iron in recurrent upper gastrointestinal bleeding.

### SWGNP Ameliorated Oxidative Stress and Energy Metabolisms Disturbed in Liver Fibrosis

**FIGURE 8:**
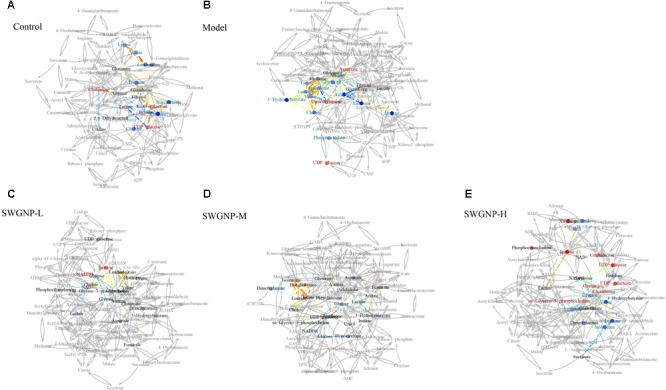
Correlation network analysis of liver tissues. **(A)** Control: Normal control group; **(B)** Model: CCl_4_-induced liver fibrosis model group; **(C)** SWGNP-L: Low dose of SWGNP treatment group; **(D)** SWGNP-M: Medium dose of SWGNP treatment group; **(E)** SWGNP-H: High dose of SWGNP treatment group. NMR correlation was connected by dotted lines, colored according to Pearson correlation coefficient and auxiliary biochemical reaction by gray solid lines. Metabolites in red and blue represented significant increase or decrease. Circles were filled by the colors according to corresponding fold changes.

**FIGURE 9:**
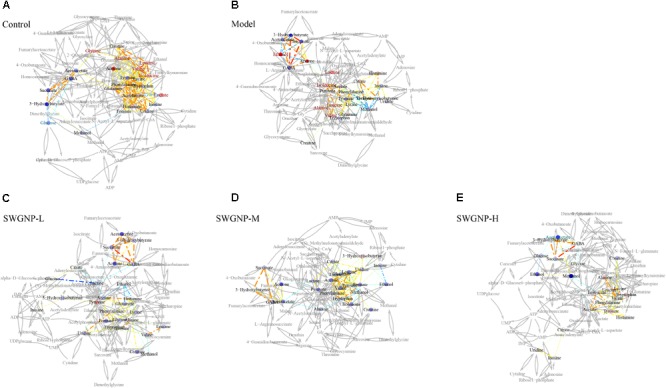
Correlation network analysis of serum from control, model and three SWGNP treated groups. **(A)** Control: Normal control group; **(B)** Model: CCl_4_-induced liver fibrosis model group; **(C)** SWGNP-L: Low dose of SWGNP treatment group; **(D)** SWGNP-M: Medium dose of SWGNP treatment group; **(E)** SWGNP-H: High dose of SWGNP treatment group. NMR correlation was connected by dotted lines, colored according to Pearson correlation coefficient and auxiliary biochemical reaction by gray solid lines. Metabolites in red and blue represented significant increase or decrease. Circles were filled by the colors according to corresponding fold changes.

The liver is the largest metabolically active organ containing a powerful antioxidant enzyme system which is important in the detoxification of external agents. In the correlation networks of control group of liver tissues (**Figure 8**), glutathione was located at the center of the metabolic network and connected with many other metabolites. In liver of fibrosis rats, by instead, glucose at the center of the network showed negative correlation with lactate, indicating the shift from aerobic respiration to aerobic oxidation. As a component of glutathione, glycine was positively correlated with glutathione, indicating that glycine was mobilized to replenish the depleted glutathione. Interestingly, lactate was located in the center of the correlation network of serum for fibrosis rats, and negatively correlated with the ketone bodies (acetone, acetoacetate and 3-hydroxybutyrate), which indicated that ketone bodies partially replaced glucose as a source of energy in the energy-deficient fibrosis rats (**Figure 9**). After SWGNP treatment, the abnormal correlations in the liver and serum of the fibrosis rats could be partially rectified. Correlation networks analysis indicated that oxidative stress and energy metabolism were the key metabolic pathways underlying the treatment effects of SWGNP.

## Discussion

In this work, a ^1^H NMR-based metabolomics approach combined with histopathological assays, elements, and biochemical parameters measurement was employed to investigate the protection of SWGNP on CCl_4_-induced liver fibrosis rats. OPLS-DA and univariate statistics as well as correlation networks analysis uncovered that SWGNP could protect rats from CCl_4_-induced liver injuries by anti-oxidation, ameliorating energy supply and improving the disordered amino acids and nucleic acids metabolisms.

### Oxidation Stress

Phosphoethanolamine, phosphocholine, and *sn*-glycero-3-phosphocholine were significantly elevated in liver fibrosis rats. Phosphoethanolamine and phosphocholine are vital components of the phospholipids in cell membranes (van Meer et al., 2008; Carman and Han, 2011). *sn*-Glycero-3-phosphocholine, a glycerophospholipid, is ubiquitous in nature and is also a key component of the lipid bilayer. As important intermediates involved in the synthesis of the characteristic bilayer structure of cells and maintenance of membrane integrity, their marked increases reflected cell membrane damage under severe oxidative stress. Oxidative stress is a major mechanism in the pathogenesis of CCl_4_-induced hepatic fibrosis in rats (Tipoe et al., 2010). The toxicity of CCl_4_ is mediated by metabolic activation by generating highly reactive trichloromethyl free radicals, leading to peroxidation of DNA, proteins, and cellular lipids.

The level of glutathione was markedly elevated in liver fibrosis rats. Glutathione is a major antioxidant which combats oxidative stress and protects the macromolecules and cell membranes from free radical damage under the help of GPX and glutathione reductase (Ceballos-Picot et al., 1996). Moreover, serum biochemical parameters indicated that large amounts of free radicals generated from CCl_4_ disturbed the levels of MDA and the activities of SOD, CAT, and GPX, which could be remarkably restored after SWGNP treatment. The increased level of glutathione in the model group could help organisms to remove the free radicals generated by CCl_4_ stress, playing a self-protective role against oxidative stress. Glutathione is derived from three amino acids (cysteine, glutamate, and glycine). The levels of glutamate and glycine in liver tissues were increased after CCl_4_ treatment. It could be inferred that their increase may account for the increased glutathione, indicating a compensation mechanism for glutathione production in fighting against free radicals. SWGNP treatment could further increase the level of glutathione, indicating its capacity in preventing oxidative injuries. Taken together, these findings indicate that SWGNP could alleviate liver fibrosis by anti-oxidation.

### Energy Metabolism

The level of glucose was significantly decreased in the liver and serum, and the level of lactate was significantly increased in the liver of fibrosis rats, suggesting a switch from mitochondrial respiration to cytosolic aerobic glycolysis within the fibrotic liver. The levels of fumarate and NADPH were obviously decreased and succinate was significantly increased in model group compared with those in the control group. Fumarate, succinate, and NADPH are intermediates of TCA cycle and/or respiratory chain. Their disorders in liver fibrosis rats indicated that TCA cycle and energy metabolism in liver mitochondria was impaired. CCl_4_ caused oxidative stress and destruction of mitochondria, resulting in the insufficiency of adenosine triphosphate (ATP) and the accumulation of lactate. Therefore, the disturbed levels of glucose, lactate, fumarate, succinate, and NADPH reflected a metabolic remodeling in response to CCl_4_ stress.

The level of acetate was significantly increased in serum of liver fibrosis rats. As the final product of fatty acids degradation (Bernson and Nicholls, 1974), the accumulation of acetate indicated that fatty acid β-oxidation was accelerated. Furthermore, markedly decreased levels of 3-hydroxybutyrate were observed in livers of CCl_4_ dosed rats. As one of the ketone bodies, 3-hydroxybutyrate has been demonstrated as an alternate source of energy which could do a favor for the organisms in case of glucose supply depletion and energy crisis occurring. CCl_4_ injection induced severe oxidative damage to hepatocytes and liver mitochondria, leading reduced glucose metabolism and deficiency of ATP generation, and the decreased generation of 3-hydroxybutyrate, produced mainly in the liver.

The levels of uridine diphosphate glucose and uridine diphosphate galactose were significantly increased in liver fibrosis rats. Uridine diphosphate glucose is an important intermediate in the process of glucose metabolism. It can be metabolized to uridine diphosphate galactose which is a precursor of glycogen that mainly deposited in the cytoplasm of the liver. The increased levels of uridine diphosphate glucose and uridine diphosphate galactose could be attributed to the accelerated glycogen decomposition to rescue the energy crisis caused by CCl_4_.

SWGNP intervention evidently decreased the levels of lactate, increased the levels of UDP-glucose and UDP-galactose, and reversed the disorders of fumarate, succinate, NADPH, acetate, and 3-hydroxybutyrate toward the normal status, indicating that SWGNP may protect against CCl_4_-induced fibrosis by regulating the perturbed energy metabolism, repairing the disordered TCA cycle, and inhibiting glycogen deposition.

### Amino Acids Metabolism

The levels of branched-chain amino acids (BCAAs) were markedly increased in serum of the fibrosis rats. BCAAs could prevent hepatic fibrosis and development of hepatocellular carcinoma (Takegoshi et al., 2017). CCl_4_-induced liver injuries could be alleviated by BCAAs through downregulating TGF-β1, Smad3, and Smad7 expressions in hepatocytes. The inhibitory effect of BCAAs on TGF-β1 signaling was mTORC1 activity-dependent. BCAAs could be more beneficial for advanced liver fibrosis patients whose serum ALB levels are decreased due to reduced mTORC1 signaling in hepatocytes (Takegoshi et al., 2017). Kawaguchi pointed out that BCAAs, particularly leucine, could activate the mTOR signaling cascade and increase ALB synthesis in animal models of cirrhosis. The levels of BCAAs were markedly increased in serum of CCl_4_ dosed rats, indicating a strong self-healing capability of liver to counteract against the toxicity of CCl_4_. SWGNP treatment could effectively prevent hepatic fibrosis and improve the antioxidant capacity of rats, making BCAAs decreased in livers of high dose of SWGNP treated rats.

Alanine was significantly increased in livers and serum of fibrosis rats. As a constituent of the glucose-alanine cycle, alanine plays an important role in the intermolecular nitrogen transport, which delivers the waste nitrogen in the skeletal muscle to the liver and metabolizes to urea (Nelson and Cox, 2013). Meanwhile, alanine can be used as a glycogenic amino acid; blood alanine could be transported to liver *via* the glucose-alanine cycle to generate pyruvate which is a source of carbon for gluconeogenesis. As CCl_4_ exposure caused severe liver damage in rats, gluconeogenesis was inhibited and glucose-alanine cycle was impaired, leading to alanine accumulation. SWGNP could effectively attenuate the increased levels of alanine, suggesting that SWGNP could rehabilitate the glucose-alanine metabolism, such playing a liver protective effect.

Glutamine was increased in serum and significantly increased in livers of fibrosis rats. Glutamine is another important amino acid for ammonia transport and nitrogen balance. Catalyzed by glutamine synthetase, blood ammonia could be transferred to glutamate to produce glutamine which then is transported into liver tissues and metabolized to urea. CCl_4_ injection caused severe liver injury as manifested by the increased serum levels of GPT and GOP, two important transaminases in liver. The accumulation of glutamine reflected an impairment of ammonium detoxication caused by CCl_4._

After SWGNP administration, the disturbed levels of amino acids in CCl_4_-dosed rats such as alanine, glutamine, glutamate, and aspartate could be reversed. The anti-fibrotic effect of SWGNP might be concerned with its modulation of the perturbed amino acids metabolisms.

### Nucleic Acids Metabolism

The levels of inosine, hypoxanthine, and xanthine were significantly decreased in livers of model group. CCl_4_ and its metabolites have the potential to damage DNA directly, or indirectly through stimulated production of reactive oxygen species, inducing genotoxicity and DNA oxidative damages in rats (Alkreathy et al., 2014). In purine catabolism, inosine can be catalyzed to hypoxanthine, which can then be catalyzed to xanthine by xanthine oxidoreductase. Xanthine can be oxidized to uric acid which is a potent antioxidant and free-radical scavenger in body. The decreased levels of inosine, hypoxanthine, and xanthine were thus helpful for the hepatic cellular to fight against CCl_4_ stress. With the improved antioxidant capacity after SWGNP treatment, the antioxidant and free-radical scavenging by nucleic acids metabolism was no longer necessary as exemplified by the elevated xanthine and inosine levels in livers of treatment rats as compared with those in the CCl_4_ dosed rats.

**FIGURE 10:**
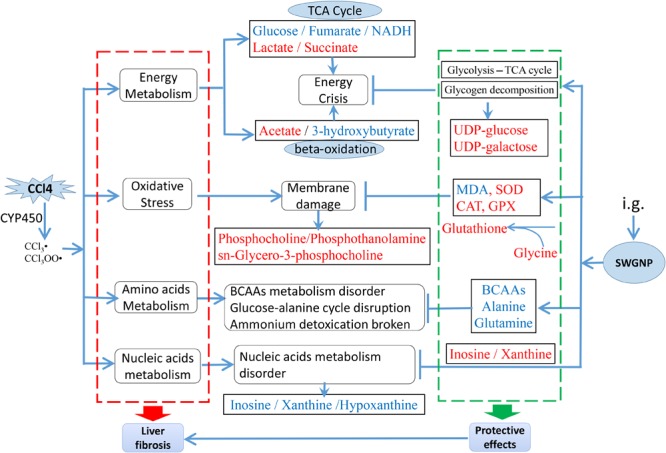
The main metabolic pathways in response to CCl_4_ induced liver fibrosis and the treatment effects of SWGNP, showing the interrelationship of the identified metabolic pathways. Metabolites in red and blue color represent significantly increase or decrease.

To sum up, CCl_4_ released excessive free radicals that evoked lipid membrane oxidation, caused oxidative stress, and shifted the energy metabolism from aerobic to anaerobic state, leading to the increase of lactate and the disorders of TCA cycle, elements metabolisms, amino acids, and nucleic acids metabolisms. SWGNP significantly decreased the level of the anaerobic product lactate and elevated the levels of compounds helpful for energy production: β-hydroxybutyrate, UDP-glucose, and UDP-galactose. SWGNP could also attenuate oxidative stress as evidenced by the significantly elevated levels of glutathione, phosphocholine, and *sn*-glycero-3-phosphocholine. The rectification of the disorders of amino acids and nucleic acids by SWGNP was demonstrated by restored levels of leucine, valine, isoleucine, xanthine, inosine, etc. A schematic diagram of the disturbed metabolic pathways was plotted, showing the interrelationship of the identified metabolites (**Figure 10**).

## Conclusion

^1^H NMR-based metabolomics approach was first applied to explore the treatment effect of SWGNP on liver fibrosis, affording prolific information to better understanding of hepatic fibrosis by identifying altered metabolic pathways. SWGNP showcased good efficacy to treat liver fibrosis according. NMR-based metabolomics approach provided a powerful and feasible tool for understanding the mechanisms underlying the efficacies of herbal formula to treat complex diseases.

## Author Contributions

XF, M-HL, J-SW, and G-JZ conceived and designed the experiments. XF, M-HL, JX, DDB, L-YR, Y-XX, and CC performed the experiments. XF, M-HL, JX, DDB, L-YR, Y-XX, CC, J-SW, and G-JZ analyzed and interpreted the data. XF, M-HL, JX, DDB, L-YR, Y-XX, CC, J-SW, G-JZ drafted and revised the work. XF, M-HL, JX, DDB, L-YR, Y-XX, CC, J-SW, and G-JZ approved the final version to be published. XF, M-HL, JX, DDB, L-YR, Y-XX, CC, J-SW, and G-JZ agreed to be accountable for all aspects of the work.

## Conflict of Interest Statement

The authors declare that the research was conducted in the absence of any commercial or financial relationships that could be construed as a potential conflict of interest.
