# Supplementary material for: Tibetan Medical Formula Shi-Wei-Gan-Ning-Pill Protects Against Carbon Tetrachloride-Induced Liver Fibrosis – An NMR-Based Metabolic Profiling
Source: Front Pharmacol. 2018 Aug 29;9:965. doi: 10.3389/fphar.2018.00965 (PMC6123542; doi:10.3389/fphar.2018.00965)
Supplement: Supplementary file 1 [file Table_1.DOCX]

Protection of Tibetan Medical Formula Shi-Wei-Gan-Ning-Pill Against Carbon Tetrachloride-Induced Liver Fibrosis Proved by NMR-Based Metabolic Profiling

Xin Feng^1,#^, Ming-Hui Li^2,#^, Jing Xia^3^, Da Ji Deng Ba^1^, Ling-Yu Ruan^2^, Yue-Xiao Xing^2^, Cheng Chen^2^, Jun-Song Wang^2,*^, Ge-Jia Zhong ^1,*^

^1^Institute for Tibetan Medicine, China Tibetology Research Center, Beijing, China.

^2^Center for Molecular Metabolism, Nanjing University of Science and Technology, Nanjing, China.

^3^Division of TCM & Natural Products/Dietary Supplement Supervisor, Shanghai Institute for Food and Drug Control, Shanghai, China.

**^#^** **The authors have the same contribution to this work.**

*** Correspondence:**Jun-Song Wang
wang.junsong@gmail.com

Ge-Jia Zhong
zhonggejia@sina.com

Keywords: Tibetan medical formula, Shi-Wei-Gan-Ning-Pill, carbon tetrachloride, liver fibrosis, NMR, metabolic profiling

**Reagents and chemicals**

Methyl alcohol (HPLC grade) was obtained from Merck KGaA (Darmstadt, Germany). Methyl alcohol and formic acid (LC-MS grade) were obtained from ROE Scientific Inc. (New York, USA).

**HPLC/DAD-Q-TOF-MS**

The methanol extract was ﬁltered through a 0.22-membrane ﬁlter before HPLC and LC-MS analysis. The HPLC analyses were performed on an Agilent 1290 HPLC instrument (Agilent Technologies Corporation, Santa Clara, CA, USA) equipped with diode array detector (DAD). Chromatographic separation was performed on Shimadzu VP-ODS column (250×4.6 mm, 5 μm particle size, Shimazdu, Kyoto, Japan), with the solvent flow rate of 1 mL/min and the temperature of 30 ºC. The injection volume was 10 μL and the detection wavelength was 210 nm, 254 nm and 280 nm. The mobile phase was composed of 0.1 % acetic acid (phase A) and methanol (phase B). The adopted solvent gradient elution was as following: 0-40 min, 10-90% B; 40-60 min, 90% B.

The positive ion ESI-MS experiments was conducted using conditions as follows: drying gas temperature, 320 ºC; drying gas (N2) flow rate, 10 L/min; nebulizer, 45 psi; capillary voltage, 4000 V for positive mode and 3500 V for negative mode; capillary current, 6.195 μA; skimmer, 65 V; fragmentor, 175 V. All the operation, acquisition, and analysis of data were made by Agilent HPLC-Q-TOF-MS MassHunter Acquisition Software Version B.04.00 (Agilent Technologies). As a result, 34 major components in the SWGNP formulae were successfully identified and were summarized in Table S1. The total ion chromatograms was showed in Figure S1.

**Table S1. Assignments of the main chemical components in SWGNP by HPLC-Q/TOF-MS.**

| **No.** | **Retation**  **Time** | **Assignment** | **Molecular**  **Formula** | **ESI-MS(+)** | |
| --- | --- | --- | --- | --- | --- |
|  |  |  |  | **Mean measured mass (m/z)** | **Error (ppm)** |
| 1 | 3.402 | herpetoriol | C_30_H_32_O_9_ | 442.1914 | 1.07 |
| 2 | 10.505 | Pirimicarb | C_11_H_18_N_4_O_2_ | 239.1488 | 6.08 |
| 3 | 12.416 | artemisinin | C_19_H_22_O_2_ | 283.1751 | 21.33 |
| 4 | 12.855 | 1,8-Diazacyclotetradecane-2,9-dione | C_12_H_22_N_2_O_2_ | 227.1750 | 2.91 |
| 5 | 14.065 | Glycinamide, 1-acetyl-L-prolyl-L-leucyl-(9CI) | C_15_H_26_N_4_O_4_ | 327.2013 | 4.1 |
| 6 | 14.749 | himalayamine | C_20_H_17_NO_6_ | 367.1056 | 0.04 |
| 7 | 15.265 | isorhoeadine | C_21_H_21_NO_6_ | 383.1364 | 0.52 |
| 8 | 15.558 | 5-Heptenoic acid, 7-[(2R,3S,4S)-tetrahydro-4,6-dihydroxy-2-[(1E,3S)-3-hydroxy-1-octen-1-yl]-2H-pyran-3-yl]-, (5Z)- | C_2_0H_3_4O_6_ | 371.2277 | -4.46 |
| 9 | 15.910 | papaverrubine | C_20_H_19_NO_6_ | 369.1216 | -0.87 |
| 10 | 16.317 | cryptopine | C_21_H_23_NO_5_ | 369.1578 | -0.42 |
| 11 | 16.849 | protopine | C_20_H_19_NO_5_ | 353.1258 | 1.55 |
| 12 | 17.5 | annuloline/dihydroberberine/ficine | C_20_H_19_NO_4_ | 337.1321 | -2.02 |
| 13 | 17.994 | menthol maltoside | C_22_H_40_O_11_ | 480.2556 | 3.13 |
| 14 | 18.998 | Sucrose, 1-laurate | C_24_H_44_O_12_ | 524.2817 | 2.92 |
| 15 | 19.910 | Barbatolic acid methyl ester | C_19_H_16_O_10_ | 405.0820 | -0.82 |
| 16 | 20.708 | Kaempferol 3-O-β-D-xylopyranosyl -(1-2)-β-D-glucopyranoside | C_26_H_28_O_15_ | 581.1509 | 1.34 |
| 17 | 23.182 | hydroxysafflor yellow A | C_27_H_30_O_15_ | 594.1589 | -0.64 |
| 18 | 23.568 | corydamine | C_20_H_18_N_2_O_4_ | 350.1268 | -0.31 |
| 19 | 24.436 | tectoridin/isotrifolin/leptosin/isoshehkanin | C_22_H_22_O_11_ | 462.1165 | 0.13 |
| 20 | 24.550 | Mesembrine/galanthusidine | C_18_H_19_NO_4_ | 313.1314 | 0.05 |
| 21 | 25.342 | ε-Isorohodomycinone | C_22_H_20_O_1_0 | 444.1049 | 1.61 |
| 22 | 31.111 | Cryptotanshinone | C_19_H_20_O_3_ | 297.1486 | -0.27 |
| 23 | 35.566 | physalin B | C_28_H_30_O_9_ | 513.2119 | -0.05 |
| 24 | 36.619 | euchroquinol A | C_16_H_18_O_3_ | 259.1309 | 7.76 |
| 25 | 37.205 | marmin monoangelate | C_24_H_30_O_6_ | 415.2125 | -2.38 |
| 26 | 38.480 | Glycocholic Acid | C_26_H_43_NO_6_ | 466.3167 | -0.01 |
| 27 | 39.414 | Acerogenin G/Auraptene | C_19_H_22_O_3_ | 299.1623 | 6.28 |
| 28 | 40.808 | dehydroemodinanthranol monomethyl ether | C_16_H_22_O_4_ | 279.1611 | -7.32 |
| 29 | 41.194 | 4',5,7-Trimethoxyflavan | C_18_H_20_O_4_ | 301.1420 | 4.91 |
| 30 | 42.393 | Allopseudocodeine/desoxycodeine | C_18_H_31_NO_3_ | 310.2381 | -1.29 |
| 31 | 43.413 | Reductodehydrocholic acid | C_24_H_36_O_5_ | 493.3150 | 3.7 |
| 32 | 47.320 | Panaxoside A | C_30_H_52_O_8_ | 541.3736 | 0.87 |
| 33 | 52.080 | 5β-Pregnane-3β,4α,5,20β-tetrol | C_21_H_36_O_4_ | 353.2667 | 5.38 |
| 34 | 57.702 | Elemic acid | C_30_H_48_O_3_ | 301.2124 | 3.32 |


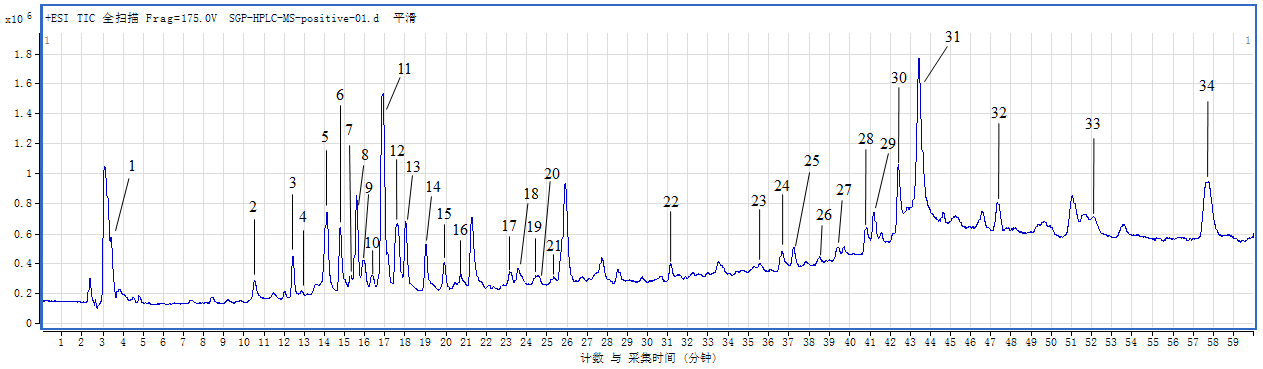


Figure S1. The total ion chromatograms of SWGNP extracts analyzed by HPLC-Q/TOF-MS
